# Clonal isolation of *Borrelia miyamotoi* identifies multiple infection phenotypes and potential novel virulence determinants

**DOI:** 10.1128/spectrum.01526-26

**Published:** 2026-10-06

**Authors:** Dawn Cleveland, Yvonne Tourand, Timothy Casselli, Trevor Long, Hoa Nguyen, Amy Aldinger, Heidi Pecoraro, Catherine A. Brissette

**Affiliations:** 1 Department of Biomedical Sciences, University of North Dakota 3579, Grand Forks, North Dakota, USA; 2 Department of Microbiology and Immunology, University of Southern Alabama 12214 , Mobile, Alabama, USA; 3 Department of Biology, University of North Dakota 3579, Grand Forks, North Dakota, USA; 4 North Dakota Veterinary Diagnostic Laboratory, North Dakota State University 3323, Fargo, North Dakota, USA; Creighton University, Omaha, Nebraska, USA

**Keywords:** *Borrelia miyamotoi*, relapsing fever

## Abstract

*Borrelia miyamotoi* is an emerging tick-borne pathogen in the Northern Hemisphere that causes a relapsing fever-like illness known as *Borrelia miyamotoi* disease. Despite increased recognition of *B. miyamotoi* as a human pathogen, the mechanisms underlying infection and pathology remain poorly understood. During routine cultivation of *B. miyamotoi* LB-2001, our lab observed that bacterial cultures extensively passaged *in vitro* (over 70 passages) lost the ability to establish infection in severe combined immunodeficiency (SCID) mice. Clonal populations were isolated from both high- and low-passage cultures, and three distinct phenotypes were emerged—infections, attenuated, and non-infectious—each showing an inverse correlation between culture growth and infectivity in SCID mice. Infection outcomes in mice revealed differences in spirochetemia, hepatosplenomegaly, and anemia among the clones. Genomic DNA sequencing identified potential virulence determinants associated with these infectious phenotypes. These findings lay the groundwork for uncovering molecular mechanisms of *B. miyamotoi* pathogenesis and identifying targets to mitigate the impact of this emerging pathogen.

**IMPORTANCE:**

As global expansion of tick habitats increases, tick-borne diseases are becoming more prevalent and widespread. The emerging tick-borne pathogen *Borrelia miyamotoi* warrants increased scrutiny. This work demonstrates loss of infectivity in immunocompromised mice by *B. miyamotoi* subjected to continual *in vitro* cultivation, as well as the isolation of clonal populations that demonstrate differences in infectivity with three distinct phenotypes: infectious, attenuated, and non-infectious. Characterizing how clonal isolates have differences in infectivity can lead to the identification of novel factors required for virulence.

## INTRODUCTION

*Borrelia miyamotoi* was first isolated in Japan in 1995 from local *Ixodes* ticks and field mice (1) though its pathogenic potential was not recognized until 16 years later when patients with acute febrile illness in Russia were found to be infected (2). Since then, *Borrelia miyamotoi* disease (BMD) has been diagnosed throughout the Northern Hemisphere—including the United States, Europe, and parts of Asia—and is now considered an emerging tick-borne disease (1, 3–11).

BMD is poorly understood, with underlying mechanisms of pathogenesis yet to be elucidated and the full spectrum of BMD symptoms still being studied. In immunocompetent individuals, BMD typically presents as a non-specific febrile illness, with symptoms such as fatigue, sleepiness, chills, muscle and joint pain, nausea, and vomiting (12–18). In immunocompromised patients, BMD often includes more severe manifestations of meningoencephalitis and neuroborreliosis (19–24). BMD has also been connected to abnormal laboratory findings related to blood coagulation and microcirculation, suggesting that *B. miyamotoi* infection can cause pathologies similar to those observed in *B. duttonii* and *B. crocidurae* infections (25, 26).

Research aimed at understanding BMD host-pathogen interactions has been limited, with challenges revolving around difficulties culturing *B. miyamotoi in vitro* and establishing infection in immunocompetent mice (27, 28). Though severe combined immunodeficiency disease (SCID) mice have utility in both respects, their extreme immunodeficiency does not fully reflect human infection (27, 29–31).

In this study, we demonstrate that multiple infection phenotypes are present in heterogeneous LB-2001. Isolation of three distinct populations—non-infectious, attenuated, and infectious—revealed an inverse correlation between *in vivo* infectivity and *in vitro* growth, corresponding with previous challenges associated with *B. miyamotoi* research. Isolated clonal populations were characterized through *in vitro* growth and *in vivo* infectivity, with cursory whole-genome sequencing. Results of this study provide a foundation for identifying novel virulence determinants and developing a murine model of *B. miyamotoi* infection.

## RESULTS

### Challenges with growth and infectivity necessitate isolating clonal populations of *B. miyamotoi*

**Fig 1:**
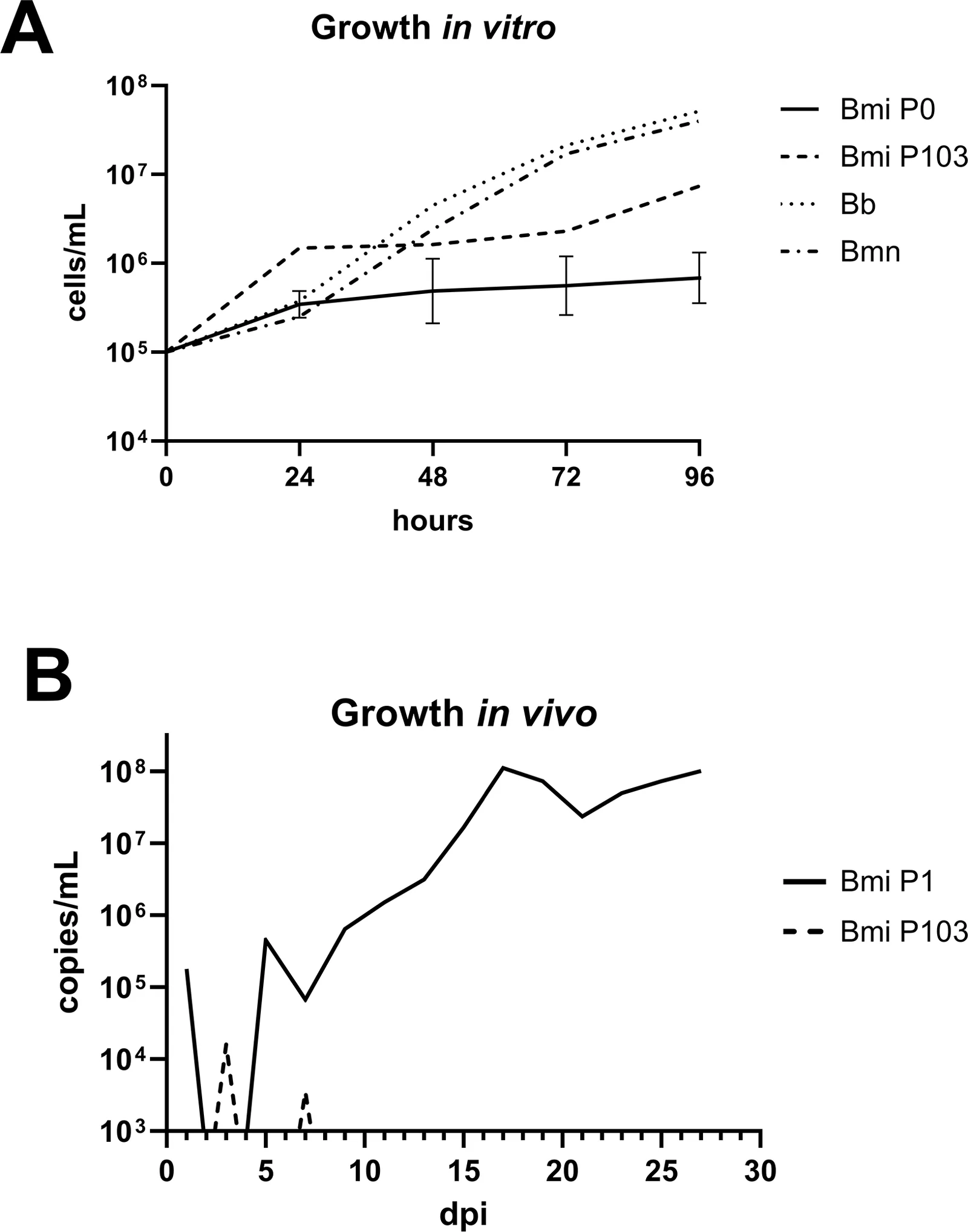
(**A**) *In vitro* growth curves of low-passage *B. miyamotoi* LB-2001 (P0, *n* = 3), high-passage *B. miyamotoi* LB-2001 (P103, *n* = 1), *B. burgdorferi* (Bb, *n* = 1), and *B. mayonii* (Bmn, *n* = 1) with the *y*-axis representing cells/mL in culture and the *x*-axis representing hours after being sub-cultured to 10^5^ cells/mL. (**B**) Spirochetemia growth curves of low-passage (P1, solid line, *n* = 1) and high-passage (P103, dashed line, *n* = 1) *B. miyamotoi* LB-2001 in the blood of SCID mice, with the *y*-axis representing qPCR-amplified copies of *glpQ* per mL of blood and the *x*-axis representing days post inoculation (dpi).

Initial attempts to grow *Borrelia miyamotoi* tick isolate LB-2001 sent to us from the Gilmore Lab at the CDC demonstrated that this isolate (passaged zero times, P0) did not grow as quickly as other *Borrelia* species we routinely cultivated, namely, *B. burgdorferi* (Bb) and *B. mayonii* (Bmn). However, the growth curve of the same *B. miyamotoi* LB-2001 isolate sub-cultured for multiple generations demonstrated growth to higher densities (passaged 103 times, P103; Fig. 1A). According to correspondence with the Gilmore Lab, the original culture should have reached densities around 10^7^ cells/mL, which was not achieved by our lab for numerous passages. The poor growth initially demonstrated may be, in part, due to differences in lab conditions or that recent revival from a freezer stock required for shipment made the culture slow-growing.

Despite poor growth in culture, low-passage *B. miyamotoi* still produced high spirochetemia in SCID mice, exceeding 10⁷ cells/mL of blood. This *in vivo* growth was determined by inoculating SCID mice, drawing blood every other day for approximately 4 weeks, and then using qPCR to quantify spirochetes in the blood. At peak spirochetemia, spirochetes could also be manually counted in serum on a Petroff-Hausser chamber. However, high-passage *B. miyamotoi* LB-2001 (P103) failed to develop high, persistent spirochetemia when inoculated into SCID mice (Fig. 1B). This contrasted with published literature (32).

To confirm infectivity differences between low- and high-passage *B. miyamotoi* cultures, it was necessary to acquire clonal populations of each phenotype. Single cells were isolated from high- and low-passage cultures via limiting dilution plating. From the high- and low- passage dilution plates, five and three clones were recovered, respectively. Clones were grown to a larger volume, made into freezer stocks, revived to make *in vitro* growth curves, and then based on growth curves, tested in SCID mice. Three distinct infection phenotypes were emerged among the clones: infectious from the low-passage plate and attenuated and non-infectious from the high-passage plate. Clone nomenclature was based on the strain of origin (LB-2001) and infection phenotype in SCID mice. Accordingly, the infectious clone is LB-V (virulent), the attenuated clone is LB-A, and the non-infectious clone is LB-S (silent).

### Relationship between *in vitro* growth and infectivity

**Fig 2:**
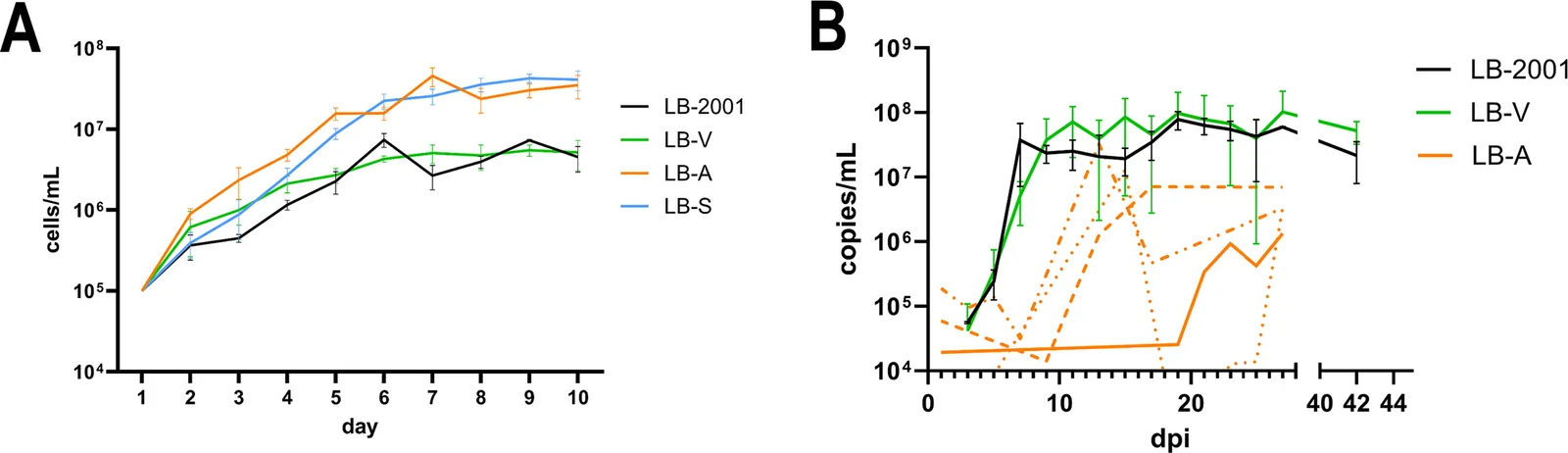
(**A**) *In vitro* growth curves of *B. miyamotoi* LB-2001 (black line), LB-V (green line), LB-A (orange line), and LB-S (blue line) (in triplicate). *X*-axis is days. *Y*-axis is cells/mL of culture. Error bars are shown. (**B**) *In vivo* spirochetemia curves of *B. miyamotoi* LB-2001 (black, *n* = 2), LB-V (green, *n* = 12), and LB-A (orange, *n* = 8). Spirochetemia curves for LB-2001 and LB-V are graphed as the mean with bars for standard deviation. LB-A spirochetemia curves are graphed from individual mice across multiple infections. Only four orange lines are visible as of the eight total SCID mice inoculated with LB-A; only four mice developed detectable levels of spirochetes in the blood, whereas the remaining four did not. *X*-axis is days post-inoculation. *Y*-axis is copies of *glpQ*/mL of blood.

An inverse correlation between *in vitro* growth and infectivity was observed for clonal *B. miyamotoi* populations, where the condition (*in vitro* or *in vivo*) with faster growth corresponded to a higher average maximum density (Fig. 2; Table 1).

**TABLE 1:** Doubling times in hours, average maximum densities with standard deviations, and number of biological replicates^*b*^

	Doubling time (hours)				Average maximum density (cells/mL × 10^6^)			
	*In vitro*	*n*	*In vivo*	*n*	*In vitro*	*n*	*In vivo*	*n*
LB-2001	21.9	3	15.5	6	5.2 ± 2.2	3	24.6 ± 15.4	6
LB-V	32.0	3	13.6	12	4.9 ± 1.3	3	66.5 ± 45.7	12
LB-A	23.3	3	30.4^*a*^	4	30.2 ± 12.8	3	4.2^*a*^ ± 7.7	4
LB-S	16.3	3	n/a	n/a	33.7 ± 10.1	3	n/a	n/a

^*a*^ Calculations only included mice that developed persistent spirochetemia (*n *= 4); doubling time was calculated during increases in spirochetemia, which varied between mice.

^*b*^ *In vitro* doubling times were calculated from 2 to 7 days. *In vivo* doubling times were calculated from 1 to 9 days for LB-2001 and LB-V. *In vivo* doubling time for LB-A was calculated during logarithmic increases in spirochetemia, which varied per animal, and was only calculated from animals that developed persistent spirochetemia. *In vitro* average maximum densities were calculated from 6 to 10 days. *In vivo* average maximum densities for LB-2001 and LB-V were calculated from 9 to 27 days when spirochetemia plateaued. *In vivo* average maximum density for LB-A was calculated once spirochetemia plateaued, which varied between animals, and was only calculated using animals that developed persistent spirochetemia. LB-S does not infect SCID mice. n/a, no growth.

Non-infectious clone LB-S exhibited the fastest *in vitro* doubling time (16.3 h) and the highest average maximum density (3.4 × 10⁷ cells/mL) among our *B. miyamotoi* isolates. Conversely, infectious clone LB-V had the slowest *in vitro* doubling time (32.0 h) and the lowest average maximum density (5.0 × 10⁶ cells/mL), taking approximately twice as long as LB-S to reach a density 10 times lower. Attenuated clone LB-A had an *in vitro* doubling time (23.3 h) and average maximum density (3.0 × 10⁷ cells/mL) that fell between the infectious and non-infectious clones. Heterogeneous LB-2001 had an *in vitro* doubling time of 21.9 h, with a maximum average density of 5.2 × 10⁶ cells/mL (Fig. 2A, C and D).

*In vivo* growth was measured by quantifying spirochetes in the blood of SCID mice over the duration of infection (Fig. 2B). This was done by inoculating groups of mice with the different strains of *B. miyamotoi*, collecting blood every other day for approximately 4 weeks, and then assessing samples for the presence of spirochetes with qPCR and culture. Mice inoculated with LB-V and LB-2001 exhibited predictable, logarithmic increases in spirochetemia (doubling times of 13.6 and 15.5 h, respectively), with peak spirochetemia (6.6 × 10⁷ and 2.5 × 10⁷ cells/mL, respectively) reached in the mice around 9 days post-inoculation (dpi) and maintained until death. In contrast, SCID mice inoculated with LB-A showed irregular, slower increases in spirochetemia (doubling time 30.4 h), taking up to 21 dpi to reach peak spirochetemia (4.2 × 10⁶ cells/mL), and only producing persistent spirochetemia in 50% of inoculated SCID mice (4/8). Despite *in vitro* success, LB-S was unable to produce infection in SCID mice (0%, 0/4) (Fig. 2B through D).

### Hepatosplenomegaly and anemia associated with *B. miyamotoi* infection in SCID mice

**Fig 3:**
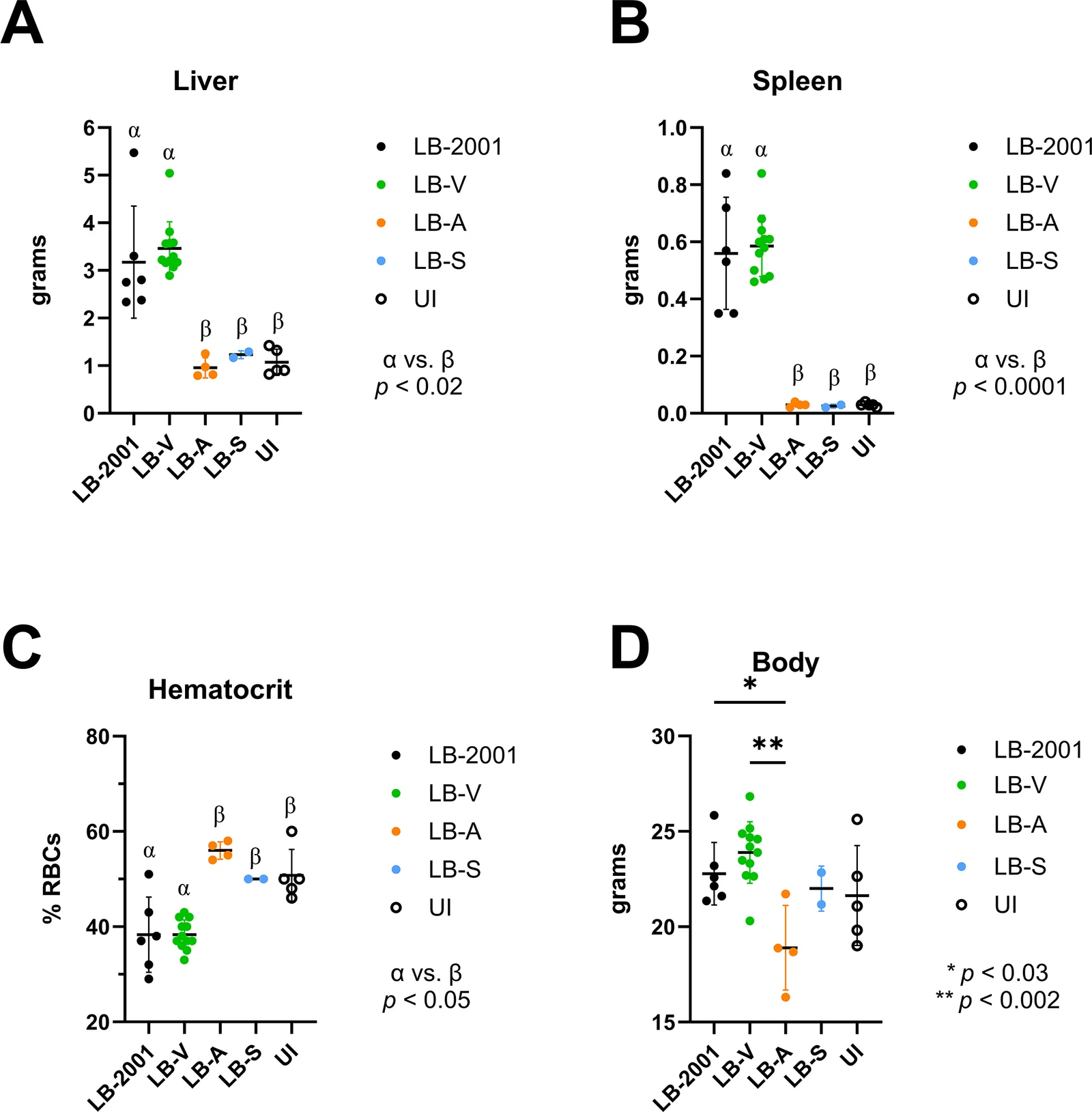
(**A**) Weight (grams) of livers and (**B**) spleens of *B. miyamotoi*-inoculated SCID mice at 6 weeks post- inoculation. (**C**) Hematocrit measured as the percentage of blood made up of red blood cells (RBCs) of *B. miyamotoi*-inoculated SCID mice at 6 weeks post- inoculation. α and β indicate statistically significant groups. (**D**) Body weight (grams) of SCID mice at 6 weeks post-inoculation prior to necropsy. LB-2001 *n* = 6; LB-V *n* = 13; LB-A *n* = 4; LB-S *n* = 2; UI *n* = 5; means and standard deviation are shown. Statistics: α vs β**:** liver (*P* < 0.02), spleen (*P* < 0.0001), hematocrit (*P* < 0.05). Body weight**:** LB-A vs LB-2001 (*P* < 0.03), LB-A vs LB-V (*P* < 0.002). *P < 0.05; **P < 0.01.

SCID mice inoculated with LB-2001 and LB-V developed hepatosplenomegaly and anemia that was statistically significant compared to matched tissues in LB-A, LB-S, and control SCID mice (UI, not inoculated with *B. miyamotoi* cultures) at 6 weeks post-infection. Livers from LB-2001- (3.17 ± 1.18 g; *n* = 6) and LB-V-inoculated (3.46 ± 0.56 g; *n* = 13) mice were on average three times larger than those from UI (1.07 ± 0.28 g; *n* = 5), LB-A (0.96 ± 0.21 g; *n* = 4), and LB-S (1.23 ± 0.08 g; *n* = 2) mice (Fig. 3A, *P* < 0.02).

Spleens from LB-2001- (0.56 ± 0.20 g; *n* = 6) and LB-V-inoculated (0.59 ± 0.11 g; *n* = 13) SCID mice were on average 19 times larger than those from UI (0.04 ± 0.01 g; *n* = 5), LB-A (0.03 ± 0.01 g; *n* = 4), and LB-S (0.03 ± 0.01 g; *n* = 2) mice (Fig. 3B, *P* < 0.0001).

LB-2001- and LB-V-inoculated mice also exhibited significantly lower hematocrit levels (38% ± 8%, *n* = 6 and 38% ± 3%, *n* = 13, respectively) compared with UI controls (51% ± 5%, *n* = 5), LB-A- (56% ± 2%, *n* = 4), and LB-S-inoculated (50% ± 0%, *n* = 2) mice (Fig. 3C, *P* < 0.05). There were no statistically significant differences in liver weight, spleen weight, or hematocrit within groups (between LB-2001 and LB-V and between LB-A, LB-S, and UI), so significance was determined using one-way ANOVA with multiple comparisons across groups for each variable.

Mice were weighed prior to necropsy. Using ordinary one-way ANOVA with Tukey’s multiple comparison test, there were no statistically significant differences between LB-2001, LB-V, LB-S, and UI mice. However, LB-A mice had significantly lower body weights compared to both LB-2001- (*P* < 0.03) and LB-V-inoculated (*P* < 0.002) mice (Fig. 3D). The reason for this is unclear. LB-A-inoculated mice were from the same litter as all LB-S-inoculated mice, two UI mice, and four of the LB-2001-inoculated mice. It is possible that LB-A has off-target effects on the host contributing to reduced body weight or that some unknown variable influenced the smaller body size.

Histopathologic analysis of livers and spleens from these animals revealed no significant differences in scoring though there was a trend toward higher liver pathology scores in LB-2001- and LB-V-inoculated SCIDs. Additionally, all tissues from LB-2001- and LB-V-inoculated mice received notes of unusual sinusoidal dilatation and congestion (Fig. S1).

### LB-2001, LB-V, and LB-A in immunocompetent C3H mice

There is limited published literature investigating *B. miyamotoi* LB-2001 in immunocompetent mice. Studies conducted in BALB/c, C3H/He(J/N), and CD-1 mice have shown that spirochetes can be detected in the blood by qPCR for up to 3 weeks post-infection, typically peaking around 10 dpi, though spirochetes are rarely visible in peripheral blood, and inoculation typically leads to the production of anti-*B*. *miyamotoi* antibodies (31–36). *B. miyamotoi* strains LB-2001, LB-V, and LB-A were assessed for infectivity in C3H mice using blood culture, qPCR, and measurements of liver, spleen, body weight, and hematocrit. LB-S was not included in these studies.

As with the SCID mice, blood was collected from C3H mice every other day for 28 days from each mouse (14 samples per mouse) for culture (qualitative) and qPCR (quantitative). Immunocompetent mice demonstrate spirochetemia much lower than in SCID mice, often below the lower limit of detection for qPCR (approximately 1,000 cells/mL of blood). Therefore, blood culture is equally important as qPCR in spirochete detection as the lower limit of detection is as few as one spirochete per blood sample.

When comparing LB-2001- (*n* = 4), LB-V- (*n* = 4), and LB-A-inoculated (*n* = 6) C3H mice, there was a significant difference in the number of positive blood cultures per mouse, as determined by Kruskal-Wallis rank analysis (*P* < 0.0001; Table 2). Of the 14 blood samples collected from each mouse, LB-2001-inoculated mice had a range of 6–7 positive cultures per mouse, LB-V-inoculated mice had a range of 3–5 positive blood cultures, and LB-A-inoculated mice had a range of 0–1 positive blood cultures (Table 2).

**TABLE 2:** Summary of C3H mice inoculated with *B. miyamotoi* LB-2001, LB-V, and LB-A^*a*^

DPI	LB-2001				LB-V				LB-A					
Mouse #	1	2	3	4	1	2	3	4	1	2	3	4	5	6
dpi														
1		+	+	+	+							+		+
3	+	+*	+	+	+*	+		+						
5		+	+*	+*			+*	+						
7	+	+*		+*	+*	+*	+*	+*						
9	+	+	+		+	+	+*							
11	+	+*	+*	+*				+						
13					+			+						
15	+*		+	+*										
17	+		+			+								
19														
21														
23		+												
25														
27	n/a	n/a	n/a	n/a	n/a	n/a	n/a	n/a						

^*a*^ + indicates positive blood culture; * indicates qPCR amplification; n/a indicates blood was not collected for qPCR or culture.

**Fig 4:**
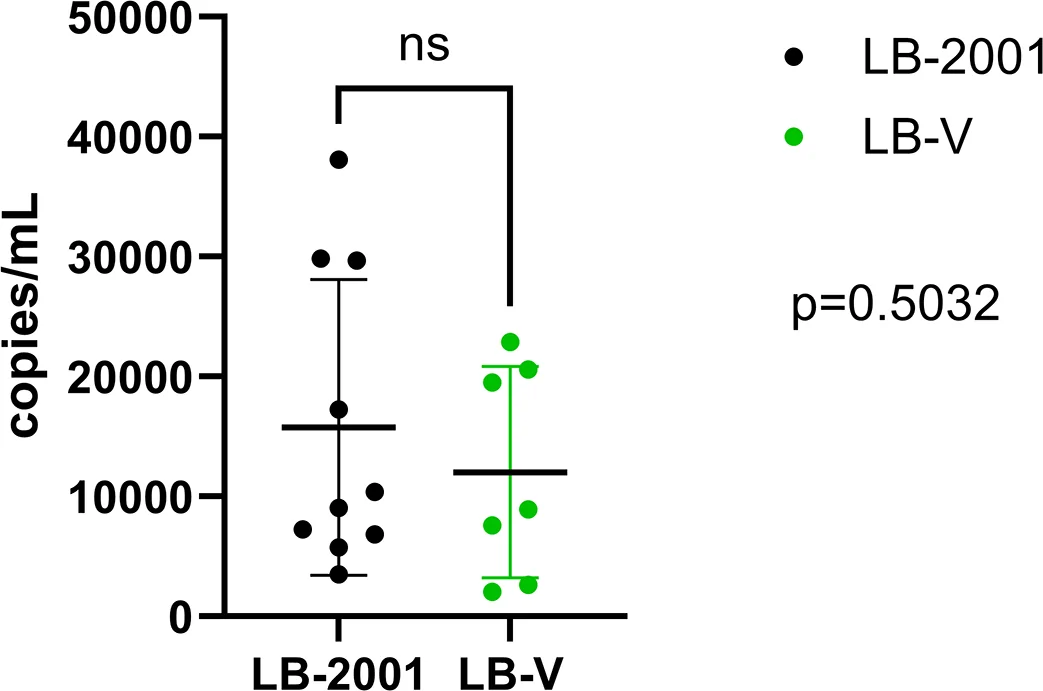
Scatter plot of quantified spirochetemia from C3H mice inoculated with *B. miyamotoi* LB-2001 (*n* = 4) or LB-V (*n* = 4) across a 4-week infection. Each dot represents *glpQ* copies amplified from one blood sample. Mean with standard deviation is shown. ns, not significant.

A similar pattern was observed for qPCR detection, detailed in Table 2: there was a significant difference in the number of qPCR-positive blood samples per mouse (*P* = 0.0002), with LB-2001-inoculated mice with a range of 1–4 blood samples with qPCR detectable spirochetemia, LB-V-inoculated mice with a range of 1–3 blood samples with qPCR detectable spirochetemia, and LB-A-inoculated mice with zero blood samples with qPCR detectable spirochetemia (Table 2). Interestingly, when comparing spirochetemia levels detected by qPCR between LB-2001- and LB-V-inoculated mice using an unpaired *t* test, there was no significant difference in bacteremia (*P* = 0.5032; Fig. 4).

**Fig 5:**
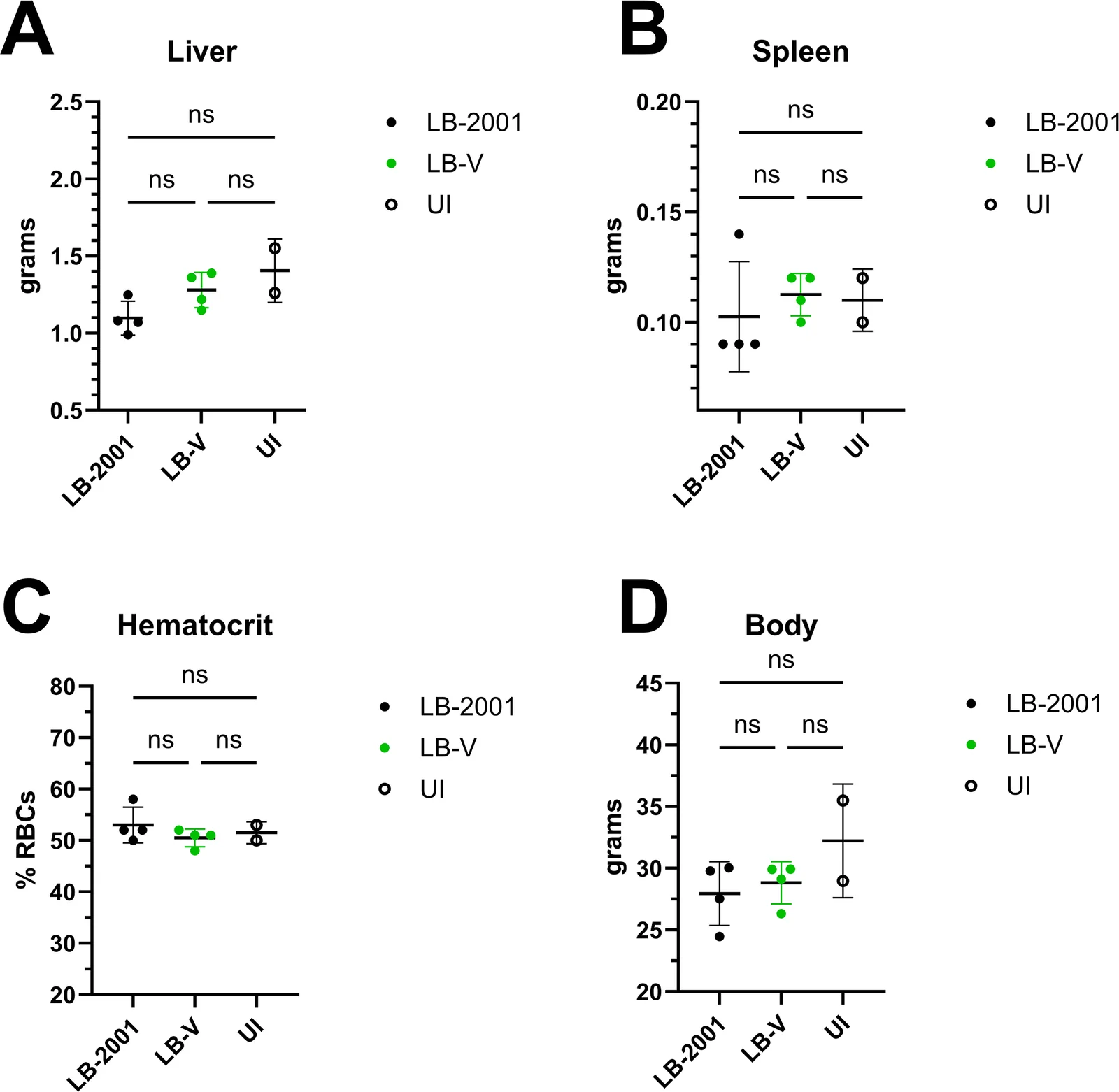
(**A**) Weight (grams) of livers and (**B**) spleens of *B. miyamotoi*-inoculated C3H mice at 6 weeks post-inoculation. (**C**) Hematocrit measured as percentage of blood made up of red blood cells (RBCs) of *B. miyamotoi*-inoculated C3H mice at 6 weeks post-inoculation. (**D**) Body weight (grams) of C3H mice at 6 weeks post-inoculation prior to necropsy. ns, not significant.

**Fig 6:**
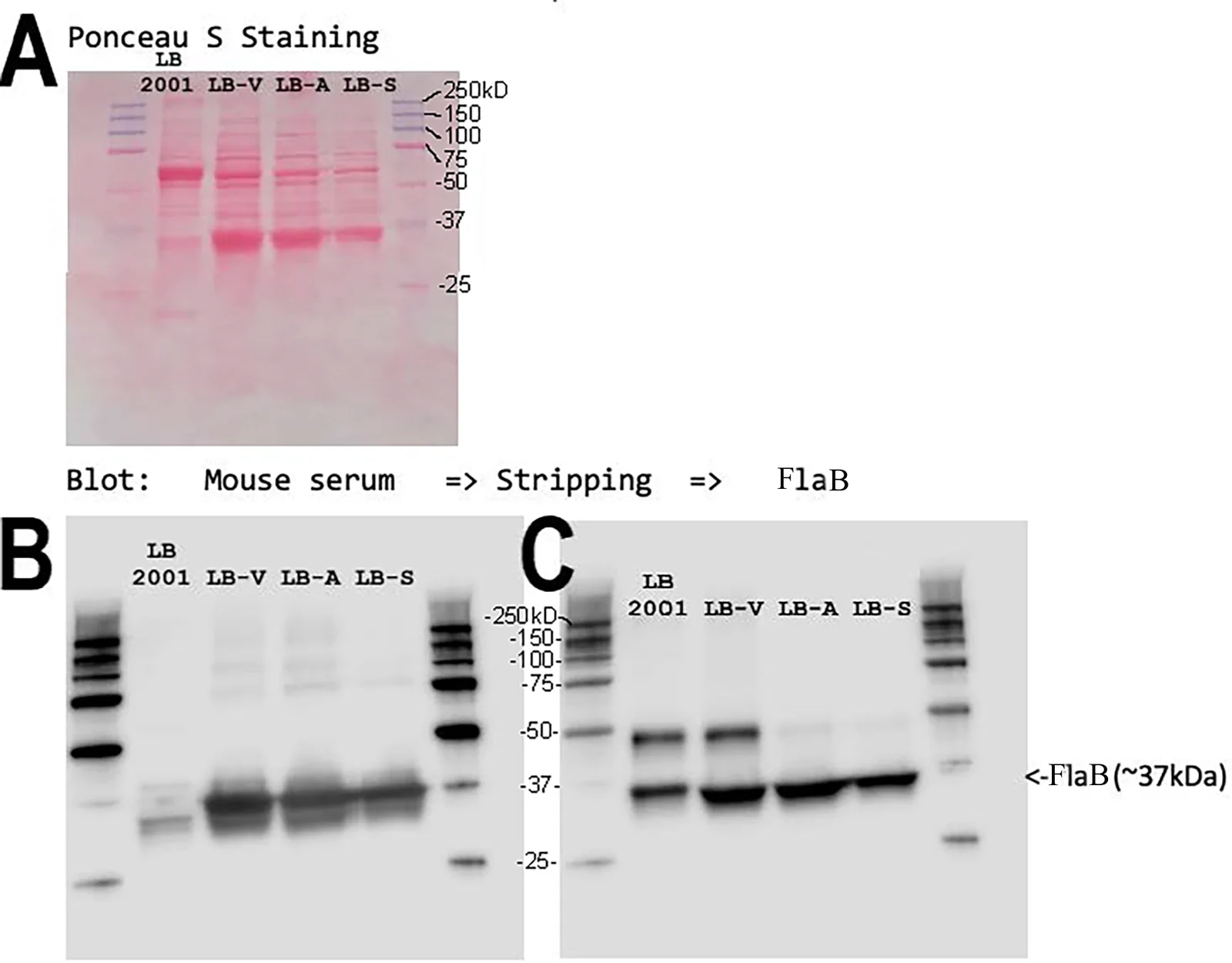
Membrane with whole cell lysate from *B. miyamotoi* LB-2001, LB-V, LB-A, and LB-S (**A**) stained with Ponceau S, (**B**) blotted with anti-*B*. *miyamotoi* serum, and (**C**) blotted with anti-*flaB* antibodies. *fla*B is approximately 37 kDa.

When liver, spleen, and body weights and hematocrit levels were compared between LB-2001-inoculated (*n* = 4), LB-V-inoculated (*n* = 4), and uninfected (*n* = 2) C3H mice at 6 weeks post-infection, there were no statistically significant differences among groups as determined by ordinary one-way ANOVA with Tukey’s multiple comparisons (Fig. 5). Seroconversion of mice inoculated with LB-2001 and LB-V was confirmed by immunoblotting using *B. miyamotoi* cell lysate, shown in Fig. 6. Mice inoculated with LB-A were not checked for seroconversion.

### Protein differences between *B. miyamotoi* isolates

Total protein differences between isolates were assessed by running whole-cell lysates of *B. miyamotoi* LB-2001, LB-V, LB-A, and LB-S on a gel and then transferred to a membrane for visualization and blotting. The membrane was initially stained with Ponceau S/Red to evaluate overall banding patterns (Fig. 6A) and then immunoblotted with anti*-B*. *miyamotoi* serum to assess different immunogenic proteins (Fig. 6B). The same membrane was then stripped and blotted with anti-FlaB antibodies as a control (Fig. 6C).

### Genomic sequencing identifies structural variants between *B. miyamotoi* clones and LB-2001 reference genome

**Fig 7:**
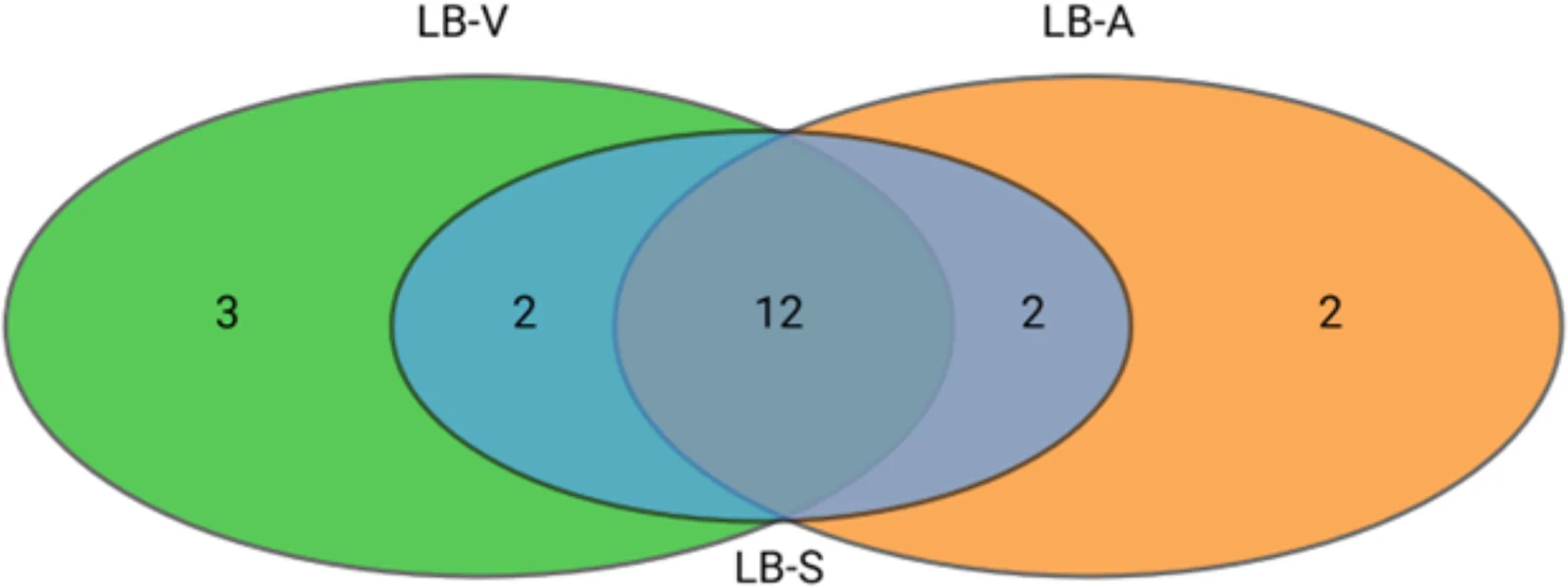
Venn diagram representing sequence variants between the *B. miyamotoi* LB-2001 reference genome and LB-V, LB-S, and LB-A.

*Borrelia miyamotoi* isolates LB-V, LB-A, and LB-S were sequenced and compared with the published LB-2001 reference genome (*B. miyamotoi* strain LB-2001, assembly GCF_000445425.4). Multiple differences were identified, with variants associated with plasmids lp41, lp31, and lp26, and the chromosome, including seven common variants shared by all clones relative to the reference genome and 14 unique variants among the clones (Table 3 and Fig. 7). These plasmids, particularly lp41 and lp26, are saturated with *vmp* genes, and most of the identified variants involve variable large family proteins (Table 3). The only variant associated with the chromosome was a deletion at position 847579 in the *infB* gene, which encodes translation initiation factor 2, unique to LB-A and LB-S (37).

**TABLE 3:** Variants associated with sequenced *B. miyamotoi* isolates LB-V, LB-A, and LB-S compared among themselves and with LB-2001 reference genome^*a*^

Plasmid	Position	Type	LB-V	LB-A	LB-S
Chromosome	847579	Deletion		X	X
**lp26**	**5854**	**Insertion**	**X**	**X**	**X**
**lp26**	**6347**	**Insertion**	**X**	**X**	**X**
lp26	8230	Insertion		X	X
lp26	8354	Deletion		X	
**lp26**	**8741**	**Deletion**	**X**	**X**	**X**
lp26	8866	Insertion		X	
**lp26**	**11437**	**Insertion**	**X**	**X**	**X**
**lp26**	**11821**	**Insertion**	**X**	**X**	**X**
*lp41*	*25428*	*Deletion*	*X*	*X*	
*lp41*	*25428*	*Insertion*			*X*
*lp41*	*25428*	*Insertion*		*X*	
*lp41*	*25428*	*Insertion*	*X*		
*lp41*	*25428*	*Insertion*	*X*		
**lp41**	**27361**	**Deletion**	**X**	**X**	**X**
**lp41**	**29954**	**Deletion**	**X**	**X**	**X**
lp26	7966	Insertion	X		
lp26	12105	Insertion	X		X
lp26	12197	Insertion	X		X
lp31	13715	Deletion	X		
lp31	16120	Deletion	X		

^*a*^ Common variants all clones have with the reference genome are in bold font and unique and partially unique variants are in regular font. Variants associated with lp41 are italicized.

## DISCUSSION

The body of research herein describes the clonal isolation of *B. miyamotoi* LB-2001 and the characterization of three unique clones: LB-V, LB-A, and LB-S. This study reports differences between clonal isolates of *B. miyamotoi* LB-2001, demonstrated throughout multiple systems that support the clonality and uniqueness of each isolate.

The kinetics of an inverse relationship between infectivity and *in vitro* growth has precedence in literature (38) and is reported for the first time in *B. miyamotoi* here. In SCID mice, infectious clone LB-V replicates rapidly in the blood, reaching the highest level of spirochetemia more quickly than any other clones. The paucity of liver- and spleen-associated pathology in SCIDs indicates that hepatosplenomegaly is likely due to high spirochetemia causing veinous obstruction and congestion (Fig. S1). In culture, conversely, non-infectious clone LB-S demonstrates the fastest doubling time and highest average maximum density, with attenuated clone LB-A an adjacent second, and LB-V in the periphery. This dynamic indicates that one or more genetic components significant for infection were lost during continual *in vitro* passage, reducing energy expenditure and allowing for more efficient replication in culture. This tradeoff may be useful to identify novel virulence determinants.

Adaptive immune pressure further distinguishes the clones from wild-type LB-2001. C3H mice inoculated with LB-2001 had significantly more blood samples with detectable spirochetes than mice inoculated with LB-V though quantified spirochetemia peaks were not significantly higher. This suggests that both LB-2001 and LB-V utilize similar mechanisms to rapidly replicate in the blood; however, heterogeneous LB-2001 is more successful at evading the adaptive immune response than clonal LB-V.

When whole-cell lysates of the *B. miyamotoi* strains were run on a gel and then blotted with anti-*B*. *miyamotoi* serum, clonal isolates demonstrated more intense banding. This could indicate that there is less variability in the proteins expressed by LB-V, LB-A, and LB-S compared to wild-type LB-2001 or that the antibodies present in the serum are more specific to proteins expressed by the clones. The anti-*B*. *miyamotoi* serum used for this blotting was an equal mix of serum collected from the C3H mice inoculated with LB-2001 and LB-V (experiments producing Fig. 4 and 5); thus, the serum used should contain both anti-LB-2001 and anti-LB-V antibodies. Ultimately, there is increased immunogenicity of LB-V, LB-A, and LB-S lysate proteins when blotted with serum from *B. miyamotoi*-infected mice compared with LB-2001 lysate.

The same gel was then stripped and blotted with anti-FlaB as a control. This demonstrated that all strains express FlaB, though at varying amounts, as all wells were loaded with the same quantity of cells. LB-2001 and LB-V also have an additional band around 48 kDa, perhaps a *B. miyamotoi* protein that is cross-reactive to FlaB.

Taken together, these data support that the LB-2001 population used in these experiments contains variability with multiple different immunogenic outer surface proteins expressed, allowing for more successful adaptive immune evasion and survival, whereas the clonal populations have reduced variability.

In immunocompetent C3H mice, there were no differences in liver and spleen sizes between LB-2001, LB-V, and uninfected mice though additional histopathological analysis and blood testing for clotting factors may reveal new details of *B. miyamotoi* infections in immunocompetent hosts. Furthermore, measurements of liver, spleen, and hematocrit were conducted at 6 weeks post-infection; had the tissues been measured during the acute phase of infection, there might have been detectable pathologies. Ultimately, the system established here can be used in future studies to elucidate host-pathogen interactions of *B. miyamotoi* in both immunocompromised and immunocompetent hosts.

Clones were sequenced and compared with the published LB-2001 reference genome. Numerous differences were associated with plasmids lp41, lp31, and lp26. Of these three plasmids, lp41 is the most well-known as it is the only identified *B. miyamotoi* virulence determinant. Lp41 encodes the *vmp* expression site, making it critical for antigenic variation and a core plasmid present across all strains (37, 39). Lp41 (also known as lp38) is approximately 38,000 bp in LB-2001. The size differences between *B. miyamotoi* strains are due to variable numbers of downstream *vmp* genes and pseudogenes, with approximately 60 distinct silent cassettes that can be translocated into the expression locus near the *pexP* promoter (37, 39, 40). This is the region at 25428 of lp41 in gray of Table 3.

Little is known about lp31 and lp26 beyond their size and presence across *B. miyamotoi* strains. Lp31 is an approximately 31,000 bp linear plasmid that also contains *vmp* genes and is found exclusively in North American *B. miyamotoi* isolates (37). Lp26 (also known as lp22) is an approximately 22,000 bp linear plasmid present across multiple continental strains including all North American, both European, and two Asian *B. miyamotoi* isolates and is saturated with *vmps* (37). Neither plasmid has been indicated in virulence. However, based on what is known of lp31 and lp26 in combination with the variants described here, these plasmids may be the location of a genetic variant or variants that change the ability to survive in a mammalian host. The main objectives of the experiments described herein were to isolate clonal populations of *B. miyamotoi* that exemplified the varying infection phenotypes demonstrated by the wild-type subjected to differing conditions (low- vs high-passage) and then to describe the differences between the clones *in vitro* and *in vivo*. Though not the primary objective of this study, the clonal isolates were sequenced to expand the breadth of knowledge for future studies, and while not definitive, the analyses conducted did offer some potential explanations for the differences between the clones. Cumulatively, these sequence variants suggest that *vmp* content contributes substantially to both the unique and shared variants observed. As not all variants involve *vmp* genes, it remains unclear whether they contribute to the differing infection phenotypes.

### Conclusion

In the isolation of clones from *B. miyamotoi* LB-2001, host- and culture-adapted strains with differing infection phenotypes were identified that demonstrate an inverse correlation between *in vitro* growth and infectivity. Further differences were characterized between clones through both *in vitro* and *in vivo* experiments, supporting the clonality of isolates and setting the foundations for future murine studies of *B. miyamotoi* host-pathogen interactions. Additionally, sequencing data of clones set the stage for the identification of novel virulence determinants associated with these infection phenotypes.

## MATERIALS AND METHODS

### Bacterial strains and culture conditions

Low-passage *B. miyamotoi* isolate LB-2001 was obtained as a gift from Dr. Bob Gilmore. Multiplex PCR screen for plasmids designed by the Gilmore lab confirmed the presence of all plasmids in low-passage LB-2001, high-passage LB-2001, and clones LB-V, LB-A, and LB-S (32). Cultures were grown to upper-log phase in MKP-F medium at 37°C, 5% CO2, and quantified by darkfield microscopy using a Petroff-Hausser counting chamber (41). *B. burgdorferi* A3 and *B. mayonii* HA growth curves were used for comparison and grown as described in Casselli et al. (42).

### Limiting dilution plating for clones

Culture growth was graphed to approximately 10^6^ cells/mL using a Petroff-Hausser chamber, and then cultures were serially diluted to concentrations of 1 cell/mL, 0.5 cells/mL, and 0.25 cells/mL in MKP-F. Each dilution was then plated on a 96-well plate with 200 µL of culture dilution per well. For the high-passage plate, the calculated volume for the dilutions allowed for plating for 64, 88, and 88 wells for the 1 cell/mL, 0.5 cells/mL, and 0.25 cells/mL dilutions, respectively. Volumes of the diluted cultures for the low-passage plate were enough to plate 96 wells for all. *Borrelia miyamotoi* does not grow to a high enough density while remaining viable to produce color change in MKP-F; therefore, each well had to be checked individually by darkfield microscopy for the presence of spirochetes. Plates were monitored for positive wells for approximately 2 months. Positive wells from the 0.25 cells/mL plates were sub-cultured into 5 mL of MKP-F and made into freezer stocks with either glycerol (culture 3:1 with 80% glycerol; final concentration 20% glycerol) or dimethyl sulfoxide (DMSO) (culture mixed 10:1 with DMSO; final concentration 10% DMSO) as a cryoprotectant.

The number of expected positive wells was calculated based on the volume of the dilution culture plated by multiplying the dilution by the volume per well by the number of wells (0.25 cells/mL × 200 µL/well × 96 wells = 4.8 cells, rounded to 5 positive expected wells). The Poisson distribution was used to determine the likelihood that positive wells from the 0.25 cells/mL plate were the result of more than one cell based on the actual number of positive wells. To calculate the probability that positive wells contain more than one cell, the sum of probabilities of more than one spirochete per well was divided by the sum of probabilities of more than zero spirochetes per well. For the high-passage 0.25 cells/mL culture plate with 5 positive wells, the probability of non-clonality is 0.0281, and for the low-passage 0.25 cells/mL culture plate, the probability of non-clonality is 0.0155. To account for the possibility that individual spirochetes may have adhered to one another during dilution, resulting in a non-clonal population, the first wells on each plate to become positive were discarded.

### Endogenous plasmid screening

The plasmid content of *B. miyamotoi* LB-2001, LB-V, LB-A, and LB-S was analyzed by multiplex PCR with specific primers designed for each of the 12 plasmids of staggered amplicon size to be visualized by gel electrophoresis (32). Larger plasmids (lp97, cp30-10) had multiple primer sets designed for different regions. Multiplex primers were designed by the Gilmore lab and conditions followed their protocol (32). PCR products were run on a 3% MetaPhor (Fisher Scientific, # BMA50181) gel run at 80 V for 180 min, followed by 35 min in ethidium bromide solution for staining, and a brief rinse. Gels were imaged using Licor Odyssey Imager Fc on 600 nm channel using Image Studio software. Some of the amplicons were not amplified in the multiplex reaction; to mitigate this, single PCR was amplified as necessary and reactions pooled for gel electrophoresis.

### Animal infections

Immunodeficient C3SnSmn.Cg-*Prkdcscic*/J (SCID; stock # 001131) and immunocompetent C3HeB/FeJ (C3H; stock # 000658) were purchased from the Jackson Laboratory (Sacramento, CA, USA). All experiments used 6- to 8-week-old female mice that were housed based on infection groups. Animals were housed and cared for at the University of North Dakota Center for Biomedical Research. Food and water were given *ad libitum*.

Mice were infected by needle-inoculation with 100 µL culture medium. For LB-2001 and LB-V, mice were inoculated with 10^5^ cells/mouse, and for LB-A and LB-S, mice were inoculated with 10^6^ cells/mouse. Differences in inoculum were due to different maximum densities between strains. Mice were anesthetized with isoflurane using VetEquip Isoflurane Vaporizer (VetEquip, # 911103), and fur was removed from the back of the neck and scapular area using electric clippers. Seventy percent ethanol was used to briefly disinfect the area. Needle-inoculation was intradermal in the upper thoracic midline. Mice were then returned to the cage and monitored until anesthesia wore off.

### Blood sample collection

Blood was collected from mice from the common iliac vein on alternating hind legs. Mice were placed head-first in a 50-mL tube with the point cut off to allow airflow. The hind leg was held straight, so hair could be shaved off the inner leg with a razor to expose the vein. The shaved area was briefly disinfected with 70% ethanol and dried; then, a needle was used to perform venipuncture. Blood was collected with a pipette as it beaded up. Gauze was used to apply pressure until clotting occurred. Approximately 20 µL of blood was collected. Exactly 10 µL of blood was added to 90 µL of SideStep Lysis and Stabilizing Buffer (“lysis buffer”; Agilent, # 400900), and the tube was flicked to mix. Approximately 10 µL of blood was added to MKP-F for blood smear or blood culture. When blood was looked at for a smear, 10 µL was added to 40 µL; this 1:5 mix was visualized using darkfield microscopy. An additional ~500 µL of MKP-F was then added to the mix for culture. Blood in lysis buffer was stored at −80°C for qPCR. Blood in MKP-F was incubated as per culture conditions. After 48 h, media were aspirated without disturbing blood cells and transferred into a fresh tube with 500–750 µL of fresh MKP-F. Cultures were checked for spirochetes using darkfield microscopy for 8 weeks after collection. Darkfield microscopy to determine the presence or absence of spirochetes in tissue culture viewed cultures at 40× and panned across the whole slide 1–3 times.

To assess infectivity, C3H mice were inoculated with LB-2001, LB-V, or LB-A, and blood was collected every other day for 4 weeks for both blood culture and qPCR. At 6 weeks post-infection, LB-2001- and LB-V-inoculated mice were euthanized, and liver, spleen, and hematocrit measurements were taken. These measurements were not performed in LB-A-inoculated C3H mice.

### Detection of *Borrelia miyamotoi* in blood using quantitative PCR

To reduce inhibition of PCR amplification due to blood products, blood in lysis buffer was column-cleaned using the Zymo *Quick*-DNA Microprep Kit according to the whole blood, plasma, and serum protocol and eluted in 20 µL elution buffer (Zymo Research, # D3021). For column-cleaning, samples were grouped by mouse with an uninfected blood sample processed alongside each group. Samples were stored at −20°C until use. qPCR was performed in 25 µL reactions with 2 µL DNA sample, *glpQ-*specific primers and probe, Bio-Rad SsoAdvanced Universal Probes Supermix (Bio-Rad, # 1725282). Forward primer CCTACAAACATTCGACTTTGATGAAT. Reverse primer CAACTTCAGCAATACCTTCTTCTGA. Probe /56-FAM/ACTGGAATG/ZEN/AAGCACCAACAGACTATGA/3IABkFQ/. Standard curves were created by amplifying known quantities of *glpQ*. Samples were quantified in triplicate, and copies per milliliter were calculated using the known quantity of blood per reaction, on the premise that one copy of *glpQ* equates to one spirochete. qPCR was validated on multiple occasions by manually counting spirochetes in plasma from an infected SCID mouse. Standards were made by using primers to amplify *glpQ* using PCR of LB-2001 (32). Zero Blunt TOPO PCR Cloning Kit (ThermoFisher, # 450245) was used to clone PCR product into *E. coli* following the manufacturer’s protocol. Plasmid DNA was purified using QIAprep Spin Miniprep Kit according to manufacturer’s protocol (Qiagen, # 27104). Copy numbers were quantified, aliquoted for standards, and stored at −80°C.

### Quantification of hepatosplenomegaly and hematocrit

SCID mice inoculated with LB-2001 (*n* = 6), LB-V (*n* = 12), LV-A (*n* = 4), LB-A (*n* = 4), and LB-S (*n* = 2), along with uninfected (*n* = 5) controls, were sacrificed at 6 weeks post-infection. Whole bodies, livers, and spleens were weighed on an electronic scale, and blood was collected for hematocrit measurement. Mice were anesthetized with inhaled isoflurane, and blood was collected via submandibular puncture while under anesthesia. Mice were euthanized by CO2 inhalation followed by cervical dislocation. Following cervical dislocation, mice were weighed on an electric scale. Livers and spleens were excised and weighed. Blood was allowed to clot at room temperature for 30 min and then centrifuged at 1,500 × *g* for 20 min. Plasma and red blood cell volumes were measured using pipettes. Spirochetes could be directly counted in the plasma of infected mice using a Petroff-Hausser Chamber.

### Histology of tissues

Livers and spleens to be used for histopathology were briefly rinsed in PBS, submerged in 40 mL of 10% neutral buffered formalin (Sigma-Aldrich, product #HT501128), and placed on a rocking plate at room temperature for 48 h with the formalin solution replaced after 24 h. Tissues were put into cassettes, mounted in paraffin, and cut on a Leica Paraffin Microtome (RM2125 RTS) in 6 µm sections. Representative sections for each sample were stained with hematoxylin and eosin for visualization with light microscopy. All histopathology scoring was done by an ACVP board-certified veterinary pathologist who was blinded to the identity of the samples. Representative images of tissues were obtained using an Epredia Pannoramic MIDI II slide scanner.

### Protein gel electrophoresis

*B. miyamotoi* cultures of LB-2001, LB-V, LB-A, and LB-S were grown to upper-log phase, counted, pelleted, treated with proteinase inhibitor cocktail (Millipore Sigma, # 5892970001), and mixed with 2× Laemmli buffer (Bio-Rad, # 1610737EDU) to a concentration of 2.5 × 10^6^ cells/µL. Samples were sonicated in a sonicating water bath for 60 s and stored at −80°C. Prior to loading gel, 1 µL of 10% β-mercaptoethanol was added to 20 µL of sample and heated at 95°C for 5 min to denature proteins. Lysate samples were loaded (20 µL/well) into a Mini-PROTEAN TGX Precast Gel (Bio-Rad, # 4561025) along with Precision Plus Protein Dual Color Standards (Bio-Rad, # 1610374) and then were run at 80 V for 10 min followed by 120 V for 1 h or until samples reached the bottom of the gel.

The gel was then transferred to a nitrocellulose membrane by layering the gel between sponges, membrane, and filter papers soaked in transfer buffer to make a sandwich, and bubbles were removed by rolling with a glass tube. The transfer sandwich was placed in a transfer cassette in cold transfer buffer and was run at 100 V for 60 min. The membrane was stained with Ponceau red for whole-cell lysate.

For Western blot analysis, the membrane was blocked with 5% milk for 1 h, rinsed 3× with TBST (Tris-buffered saline with Tween), incubated with 1:1,000 anti-*B*. *miyamotoi* serum (harvested from LB-2001- and LB-V-inoculated C3H mice; equal parts anti-LB-2001 and anti-LB-V) for 1 h, washed three times with TBST, incubated with 1:5,000 Goat anti-mouse IgG (H + L) Secondary Antibody, HRP (ThermoFisher, # 31430) for 1 h, and rinsed again times with TBST. The gel was drained and SuperSignal West Pico PLUS Chemiluminescent Substrate (ThermoFisher, # 34580) was mixed according to the manufacturer’s protocol and added to cover the membrane and then incubated for 3 min at room temperature. The membrane was imaged using the chemi channel on the Licor Odyssey Imager with an exposure time set to 2 min. The membrane was then washed with TBST and stripped with Restore PLUS Western Blot Stripping Buffer (Thermo Scientific), and the same steps were followed using anti-FlaB (Rockland, #200-401-C14).

### Genomic DNA sequencing and analysis

For DNA extraction, cultures were grown to upper-log phase, pelleted and rinsed three times with PBS, lysed with 10% SDS, and treated with RNAseA and Proteinase K. Nucleic acids were extracted using UltraPurePhenol:Chloroform:Isoamyl alcohol (25:25:1, vol/vol; ThermoFisher, # 15593031). Sample volume was brought to 200 µL with Buffer EB (Qiagen, # 19086). Twice, an equal volume of Phenol:Chloroform:Isoamyl alcohol was added to samples in 2 mL Manual Phase Lock Gel (PLG) tubes (LifeSCT, # M2302-20), samples were rotated at room temperature for 20 min, and then, the aqueous layer containing DNA was carefully aspirated. This was repeated with chloroform only. DNA was precipitated with sodium acetate and glycogen, mixed with 500 µL ethanol, and stored at −20°C overnight. Samples were centrifuged, supernatant was aspirated, and the pellet was washed with 70% ethanol, allowed to air dry, and then cleaned with AMPure XP Beads (Beckman Coulter, # A63880). DNA samples were quantified using Qubit 1× dsDNA HS Assay Kit (ThermoFisher, # Q332030) according to the manufacturer’s protocol.

Nanopore sequencing was performed by the UND Genomics core on genomic DNA from LB-V, LB-A, and LB-S. Libraries were generated under their care, and samples were run on GridION X5 Nanopore platform.

Basecalling and filtering were done by the University of North Dakota Genomics core. Filtered reads were aligned to *B. miyamotoi* LB-2001 assembly GCF_000445425.4 using Minimap2 (43) with default settings as follows: minimap2 -ax map-ont <referenceFasta> <sampleFastq>> <sample> .sam. Aligned reads were then sorted and indexed using Samtools (44), while structural variants were identified using Sniffles2 (45) as follows: sniffles -i <mapped_input.bam> --reference <referenceFasta> v <sample> .vcf. The resulting VCF files were read into R (v.4.3.0) using *vcfR* package (46), and variants were quantified using the *VennDiagram* package (47).

## Data Availability

The whole-genome sequence data for LB-V, LB-A, and LB-S generated in this study have been deposited to NCBI under BioProject accession number PRJNA1511927 and BioSample accession numbers SAMN62334060–SAMN62334062.

## SUPPLEMENTAL MATERIAL

Fig. S1 spectrum.01526-26-s0001.tif Liver histopathology.

Supplemental legend spectrum.01526-26-s0002.docx Legend for Fig. S1.

ASM does not own the copyrights to Supplemental Material that may be linked to, or accessed through, an article. The authors have granted ASM a non-exclusive, world-wide license to publish the Supplemental Material files. Please contact the corresponding author directly for reuse.
