# Supplementary material for: Clonal isolation of Borrelia miyamotoi identifies multiple infection phenotypes and potential novel virulence determinants
Source: Microbiol Spectr. 2026 Oct 6;14(11):e01526-26. doi: 10.1128/spectrum.01526-26 (PMC13729108; doi:10.1128/spectrum.01526-26)
Supplement: Supplementary file 1 [file spectrum.01526-26-s0002.docx]

Supplemental Figure Legend

Histopathology images (10x) of livers with and spleens stained with H&E from inoculated SCID mice at 6 weeks post inoculation with scoring notes from veterinary histopathologist. Mild lymphoid expansion suspected in LB-2001 and LB-V spleens as well but obscured by blood.

EMH – extramedullary hematopoiesis, normal background lesion in mice
